# Mining Trends of COVID-19 Vaccine Beliefs on Twitter With Lexical Embeddings: Longitudinal Observational Study

**DOI:** 10.2196/34315

**Published:** 2023-05-02

**Authors:** Harshita Chopra, Aniket Vashishtha, Ridam Pal, Ashima, Ananya Tyagi, Tavpritesh Sethi

**Affiliations:** 1 Guru Gobind Singh Indraprastha University New Delhi India; 2 Indraprastha Institute of Information Technology New Delhi India; 3 All India Institute of Medical Sciences New Delhi India

**Keywords:** COVID-19, COVID-19 vaccination, vaccine hesitancy, public health, unsupervised word embeddings, natural language preprocessing, social media, Twitter

## Abstract

**Background:**

Social media plays a pivotal role in disseminating news globally and acts as a platform for people to express their opinions on various topics. A wide variety of views accompany COVID-19 vaccination drives across the globe, often colored by emotions that change along with rising cases, approval of vaccines, and multiple factors discussed online.

**Objective:**

This study aims to analyze the temporal evolution of different emotions and the related influencing factors in tweets belonging to 5 countries with vital vaccine rollout programs, namely India, the United States, Brazil, the United Kingdom, and Australia.

**Methods:**

We extracted a corpus of nearly 1.8 million Twitter posts related to COVID-19 vaccination and created 2 classes of lexical categories—emotions and influencing factors. Using cosine distance from selected seed words’ embeddings, we expanded the vocabulary of each category and tracked the longitudinal change in their strength from June 2020 to April 2021 in each country. Community detection algorithms were used to find modules in positive correlation networks.

**Results:**

Our findings indicated the varying relationship among emotions and influencing factors across countries. Tweets expressing hesitancy toward vaccines represented the highest mentions of health-related effects in all countries, which reduced from 41% to 39% in India. We also observed a significant change (*P*<.001) in the linear trends of categories like hesitation and contentment before and after approval of vaccines. After the vaccine approval, 42% of tweets coming from India and 45% of tweets from the United States represented the “vaccine_rollout” category. Negative emotions like rage and sorrow gained the highest importance in the alluvial diagram and formed a significant module with all the influencing factors in April 2021, when India observed the second wave of COVID-19 cases.

**Conclusions:**

By extracting and visualizing these tweets, we propose that such a framework may help guide the design of effective vaccine campaigns and be used by policy makers to model vaccine uptake and targeted interventions.

## Introduction

The unprecedented spread of COVID-19 has created massive turmoil in public health around the world [1]. The development of vaccines has played a pivotal role in eradicating and mitigating significant outbreaks of infectious diseases like smallpox, tuberculosis, measles, and similar contagious diseases [2]. Major pharmaceutical companies located across the globe are in the phase of developing vaccines, with only a handful of the vaccines authorized for clinical trials [3,4]. As the distribution of vaccines and associated campaigns expand, people continue to express their opinions and personal incidents on social media platforms.

Social media plays a decisive role in propagating information, leading to the emergence of varying perceptions related to the pandemic [5]. During the initial phase of national lockdown in several countries, Twitter had reported an increase of 24% in daily active users due to the increased usage of social media, the highest year-over-year growth rate reported by the company to date [6].

Mass media strongly influences vaccine uptake and vaccination rates, as shown previously for influenza [7,8]. Although some studies have also shown a positive impact of mass media on improving vaccine uptake and mitigating hesitancy [9], its role in the spread of vaccine misinformation and conspiracy theories has been widespread [10]. Recent studies such as “The ‘Pandemic’ of Disinformation in COVID-19” [11] reported several events for which mass media channels have misinformed the public by sharing incomplete or unverified updates on new treatments, myths about usage of masks, and errors of some hospital organizations that resulted in higher reluctance from patients to go to hospitals or medical centres. The surge in consumption of COVID-19 updates from mass media channels has impacted different age groups by inducing panic and anxiety [12].

The COVID-19 pandemic has been studied in multidisciplinary aspects, and the analysis of Twitter posts remains a widely explored area in public health research [13-15], primarily because of the rapidly evolving nature of the content. Over the last decade, researchers have used multiple methods such as sentiment classification [16], social network analysis [17], and topic identification [18] to study the presence of provaccine and antivaccine communities on social media. It has been observed that vaccine uptake is affected by multiple factors, including rising adverse effect reporting, socioeconomic inequities, and quantitative allocation [19]. In addition, the spread of misinformation online has been a concerning issue, and prior survey-based studies suggest that it is linked with vaccine hesitancy and effects on public health [20,21]. On the other hand, certain marginalized groups continue to face inaccessibility to vaccines [22].

This paper presents a temporal and demographic analysis of lexical categories mined from Twitter conversations around vaccines. We further subdivided these categories into 2 subtypes: emotions and their influencing factors. We examined the relationships between emotions such as hesitancy, rage, contentment, sorrow, faith, and anticipation with influencing factors such as conspiracy theories around vaccines, social inequities, and health effects using unsupervised word embeddings trained on the curated corpus of tweets during an 11-month period. Further, we created correlation-based networks of these categories and performed clustering using the Infomap algorithm. The alluvial diagrams generated by these networks demonstrate the flow of importance of each factor from one month to another. We performed a granular analysis of the temporal-based trends of various outlooks toward COVID-19 vaccine activities. We analyzed their correlation with prominent factors for 5 countries (India, the United States, Brazil, the United Kingdom, and Australia) located on 5 different continents to demonstrate the comparative results among them.

Recent research work has analyzed vaccine hesitancy or sentiment analysis to determine the overall general perception among people toward COVID-19 vaccines. Our work provides a more detailed insight into the variety of outlooks people had toward the emergence of continuous vaccine updates and possible correlations with reasons for these outlooks. Major analysis work on survey data in specific regions or a cohort of the population has helped understand people’s opinions toward vaccine uptake or resistance. Still, we have worked on a large corpus of tweets (more than 1.8 million) from different countries. As the meteoric rise in the use of social media has become a substantial influencing source for formulating different perceptions in millions of users, working with such a data source helps gain a broader and better sense of various factors that might be associated with fueling vaccine resistance. We have also analyzed our findings with vaccine developments and news in each country during the specific time periods to support our results.

## Methods

### Design and Data Set

We performed an observational study by curating a longitudinal data set by scraping more than 1.8 million tweets using the Snscrape library [23] from June 2020 to April 2021. The query used to extract the tweets was created using an “OR” combination of hashtags and words related to vaccines and the names of the vaccines administered in the respective countries. Detailed queries for each country are mentioned in Table 1.

**Figure 1:**
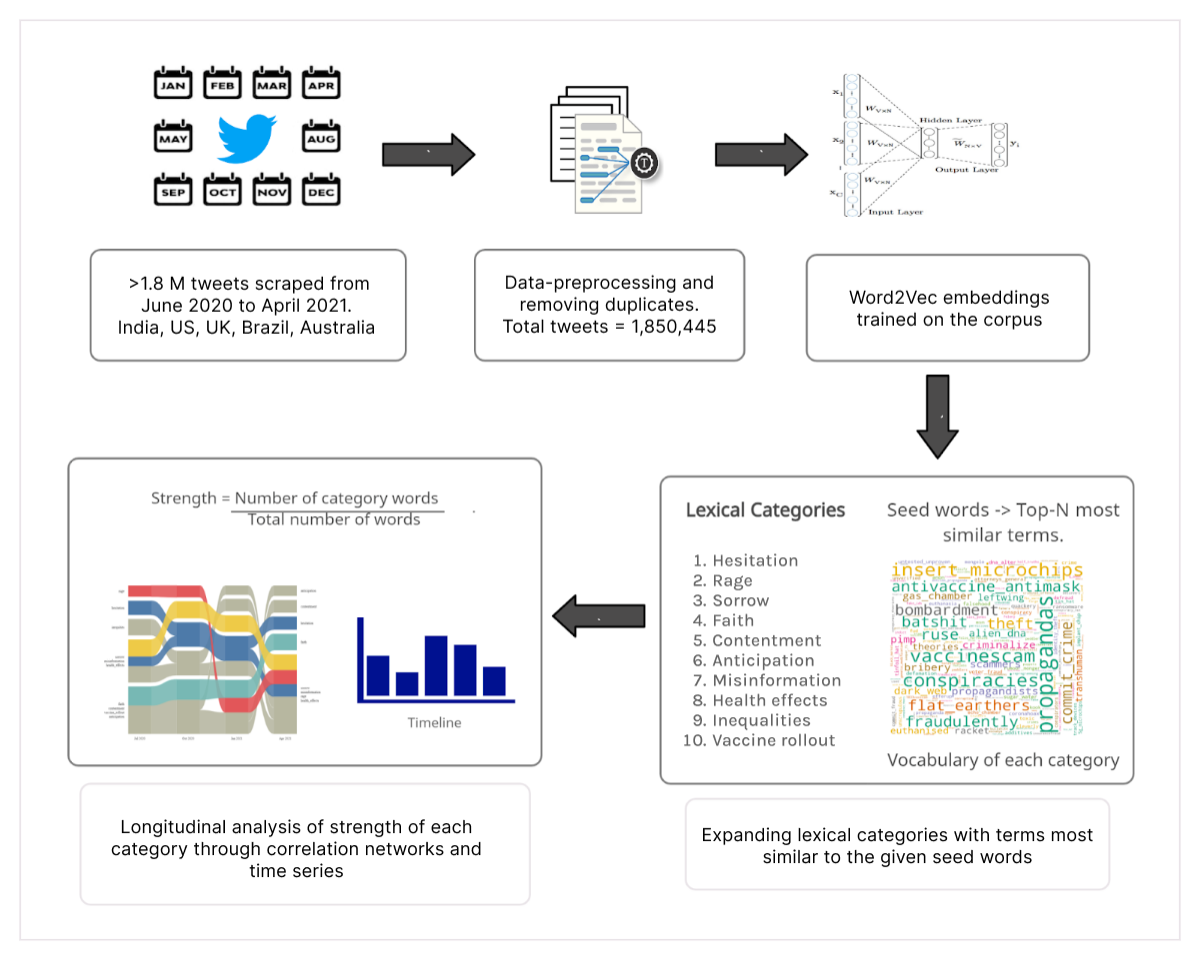
Overview of the pipeline followed to create and analyze the strength of lexical categories.

Preprocessing of tweets was carried out on lowercase-converted text by removing white spaces, punctuation, hashtags, mentions, digits, stop words, URLs, and HTML characters. The verbs present in the text were lemmatized using WordNet Lemmatizer from the Natural Language Toolkit (NLTK) package [24]. Duplicate tweets were removed based on identical username, time, and location. Figure 1 illustrates an abstract view of the study design. We list all the software and packages used in further analysis along with the corresponding versions and sources in Multimedia Appendix 1.

**Table 1:** Queries used for scraping tweets from each country and number of tweets used after preprocessing.

Country	Query^a^	Tweets, n
United States	(General keywords) OR (moderna OR pfizer OR biontech OR astrazeneca OR inovio OR novavax OR #pfizerbiontech)	1,121,216
United Kingdom	(General keywords) OR (pfizer OR biontech OR oxfordvaccine OR astrazeneca OR moderna OR #pfizerbiontech)	432,271
India	(General keywords) OR (covishield OR covaxin)	229,127
Australia	(General keywords) OR (pfizer OR biontech OR oxfordvaccine OR astrazeneca OR moderna OR novavax OR #pfizerbiontech)	50,224
Brazil	(General keywords) OR (coronavac OR Sinovac OR AstraZeneca OR Pfizer OR BioNTech OR #pfizerbiontech OR oxfordvaccine)	17,608

^a^General keywords: (vaccine OR vaccination OR vaccinate OR covax OR #covidvaccine OR #coronavaccine OR #covidvaccination).

### Ethics Approval

Publicly available Twitter data were used, and an aggregated analysis was performed without any attempt to re-identify or link any personal information. The study received institutional review board approval (IIITD/IEC/08/2021-6) and was conducted under the oversight of the associated protocol.

### Curating Categories Using Unsupervised Word Embeddings

We created 10 lexical categories for a psychometric evaluation of the tweet content in an approach similar to that by Empath [25]. The categories formed can be broken down into 2 classes: “emotions” and “influencing factors.” Emotions consist of the affective processes that help us understand how reactions, feelings, thoughts, and behavior of people evolve in a given situation. We selected 6 COVID-19–related emotions, namely hesitation, rage, sorrow, faith, contentment, and anticipation, along with their putative influencing factors such as misinformation, vaccine rollout, inequities, and health effects in contrast to the COVID-19 vaccines. We specified a set of seed words corresponding to these categories, as shown in Table 2.

We trained a low dimensional representation (d=100) as word embeddings for the unigrams and frequently occurring bigrams (co-occurring at least 5 times with the bigram scoring function [26] greater than a threshold of 50) present in our corpus using the skip-gram algorithm of the Word2Vec model [27] with a sliding window size of 5. We defined lexical categories as sets of words most similar to the assigned seed words. Each seed word, ensured to be present in the model’s vocabulary, was mapped to a word vector. We used cosine similarity to measure proximity to find the top N(=50) words in the nearby vector space. Following this approach, k seed words were expanded to a list of maximum k×N words. A category was defined as the union set of seed words and their closest similar words (Table 2). Seed words used for the health effects category were taken from the adverse events mentioned in the Vaccine Adverse Event Reporting System (VAERS) database [28], which occurred in our data set’s vocabulary. The resulting set of words in each lexical category was manually verified.

**Table 2:** Curated categories (emotions and influencing factors), their description, and seed words.

Category			Description		Seed words
**Emotions**					
	1. Hesitation	Sceptic attitude and reluctance toward being vaccinated due to multiple negative factors affecting an individual’s opinions		Anxious, nervous, fear, consequences, uncertain, hesitation, suspicion, harm	
	2. Sorrow	Dissatisfaction and disapproval toward the different phases of COVID-19 vaccine production and distribution		Sad, hopeless, worst, disappointment, setback	
	3. Faith	Signifies strong belief and confidence in vaccines along with optimistic behavior toward the success of vaccines		Faith, optimism, vaccines work, assurance, grateful	
	4. Contentment	Signifies a state of happiness, appreciation, and acceptance of the COVID-19 vaccines		Satisfy, glad, proud, gratitude, great, joy	
	5. Anticipation	State of urgent demand and necessity of vaccines		Anticipate, urgently, priority, quick, await	
	6. Rage	Anger or aggression is associated with conflict arising from a particular situation		Angry, annoyance, hate, mad, pathetic	
**Influencing factors**					
	7. Misinformation	Propagation of false information such as misinterpreted agendas and conceiving vaccines as conspiracy or scam		Propaganda, conspiracy, fraud, fake, poison	
	8. Vaccine rollout	Availability and distribution of vaccines through campaigns and mass vaccination drives		Vaccinate, distribution, supply, mass, dose, vaccination drive	
	9. Inequities	Socioeconomic disparities are based on societal norms such as caste, race, religion		Socioeconomic, deprive, racial injustice, racism, underrepresented	
	10. Health effects	Mentions of health-related adverse events caused by or affected by vaccines, including diseases, symptoms, and pre-existing conditions		From the VAERS^a^ database (eg, headache, fatigue, inflammation)	

^a^VAERS: Vaccine Adverse Event Reporting System.

### Temporal Analysis of Lexical Categories

To measure each category’s strength in a given text, we used the word count approach, similar to that by Empath [25] and other lexicon-based tools like Linguistic Inquiry and Word Count (LIWC) [29]. To obtain an unbiased value that is independent of the length of text, we divided the frequency by the total number of words using the following formula:



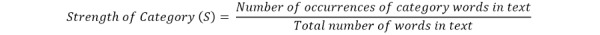



We appended the preprocessed text of all tweets monthly to calculate the strength. The time series of the strength of emotion categories and influencing factors was helpful in analyzing the evolution of perceptions and opinions expressed by the public and how they vary with crucial time stamps like the news of the country’s first vaccine approval.

### Analysis of Change Before and After Approval

To understand the variation of emotions among social media users in the aftermath of the approval of vaccines, we conducted a before-after change analysis for each lexical category based on the date when the country’s government approved the first COVID-19 vaccine.

We created a day-wise time series of the strength of each category from June 2020 to April 2021 and smoothened it using the Moving Average algorithm. The linear nature of the trend was captured using an ordinary linear regression model fit on the strength of a category in the 2 time periods preceding and succeeding the approval date. To calculate the significance of the change, we used the *z* test to compare the regression coefficients [30]:



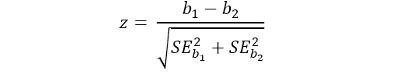



where *b*_1_ and *b*_2_ denote the slopes and 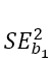 and 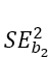 are the standard errors of the regression lines and before and after the approval, respectively.

Further, we used a change-point detection method based on dynamic programming using the Ruptures package [31] in Python3. The “Dynp” model was used with the “l1” cost function to detect one change point. This was done to verify if the date of approval was close to the change point.

To understand the Influencing factors co-occurring with hesitation, we resampled the tweets with a positive strength of hesitation (n=1000) and calculated the percentage of tweets that also had positive strength of anticipation, rage, misinformation, health effects, and inequities. The resampling was repeated for 100 iterations, and the mean and standard errors were plotted. The percentages of tweets from each of these categories that changed before and after the approval were recorded and tested for significance.

### Longitudinal Correlation-Based Networks

The correlation between any 2 categories represents the degree to which they are linearly related. Daily strengths were calculated for each category followed by pairwise Pearson correlation [32]. Weighted networks of categories (nodes) and edge strengths (correlation coefficients) were constructed to evaluate the positive associations among classes (*ρ*≥0). Community detection on these networks was carried out using the Infomap algorithm [33], and the dynamic change in these associations was visualized as an alluvial diagram [34]. The use of the Pearson correlation typically requires the verification of some assumptions. We verified the assumption of outliers by plotting box plots of the samples and observed very few or no outliers. To check for a normal distribution, we used the Shapiro-Wilk test (used for n_samples<50), which was satisfied for most but not all months. Hence, we also present the analyses using Spearman correlation, a nonparametric measure, to construct the alluvial diagrams, as shown in Figure S1 in Multimedia Appendix 2.

## Results

### Analysis of Lexical Categories

Unsupervised word embeddings capture the context of words in the latent space based on their distribution and patterns of co-occurrence [35]. Given the noisy nature of social media data, it becomes difficult to implement a predefined lexicon-based approach with appropriate semantic inclusion. In this paper, we used unsupervised word embeddings trained on our corpus of tweets to find the words most similar to a given set of seed words, hence expanding the vocabulary of a lexical category. Table 3 shows the words belonging to the categories of hesitation and misinformation. The lexical category of hesitation represents words such as “skeptical,” “disillusionment,” “needle-phobic,” “dissonance,” and “consequence,” which demonstrate the uncertainty and doubt regarding vaccines and their effects. Some of the words most similar to “conspiracy” were found to be “implant_microchips” (cosθ=0.844), “qanon_conspiracy” (cosθ=0.820), “tinfoil_hat” (cosθ=0.808), and “echo_chamber” (cosθ=0.806). These terms denote how people link vaccines to unconventional concepts and propaganda.

**Table 3:** Words belonging to the lexical categories of hesitation and misinformation, representing the vocabulary expanded from the seed words of the respective categories.

Category	Category words
Hesitation	Confusions, trade_off, shortterm_longterm, frustrate, damage, popularize, apprehension, notions, tire, harmful
Misinformation	Frenzy, propaganda, lethal_injection, false_narratives, black_ market, insert_microchips, euthanised, unsafe_untested, non_believers, conspiracy_theory

### Change in Trends Before and After Approval

**Figure 2:**
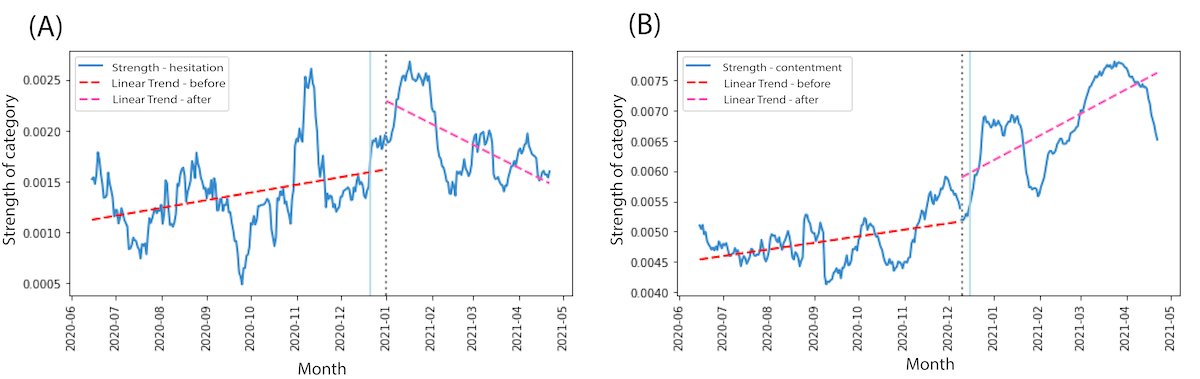
Linear variation in the strength of (A) hesitation in India and (B) contentment in the United States. The dotted line represents the date of approval, and the light blue line depicts the detected change point.

The difference in slopes of the linear trends of the before and after periods for each category demonstrate 2 significant inferences: the magnitude of change and the direction of change. Figure 2A shows the trends for hesitation in India. A significant change in the direction of the slope is evident (*z*=10.37, *P*<.001), which depicts a decrease in its strength after the approval. There was a significant increase (*z*=–7.65, *P*<.001) in the magnitude of tweets expressing contentment during the vaccination phase in the United States as shown in Figure 2B. The detected change point was found to be lying within the ranges of 6 days (Figure 2A) and 10 days (Figure 2B) of the date of approval.

**Figure 3:**
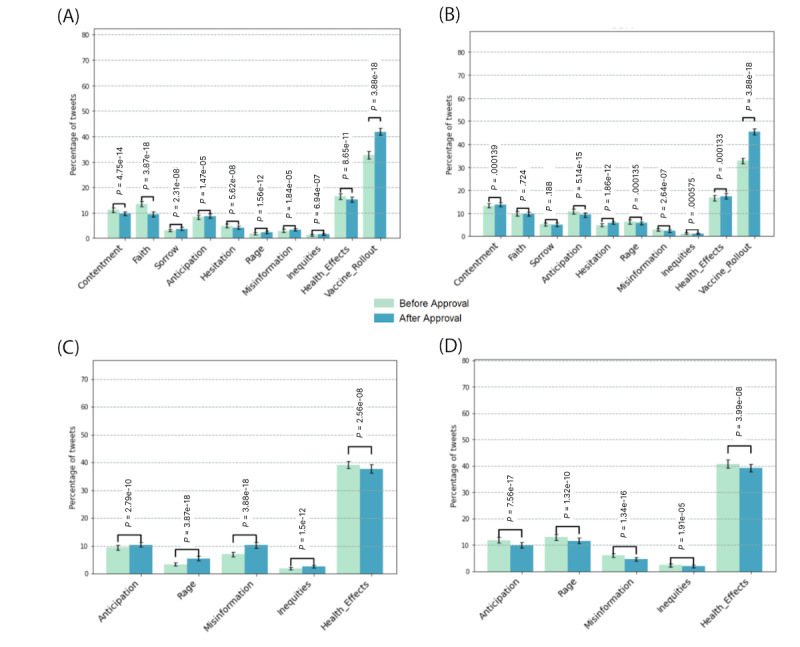
Percentage of tweets with a positive strength in each lexical category before and after approval of COVID-19 vaccine in (A) India (January 1, 2021) and (B) the United States (December 10, 2020) and the percentage of anticipation, rage, misinformation, inequities, and health effects in positive “hesitancy” tweets in (C) India and (D) the United States.

The percentage of tweets belonging to different categories was analyzed from the sample of tweets before and after the approval of vaccines in each country. Figure 3A shows that faith and contentment were both significantly higher (both *P*<.001) before the approval of the first vaccine in India on January 01, 2021 [36]. The factors co-occurring with hesitation were analyzed by calculating the percentage of tweets of 5 other categories (Figures 3C and 3D). Our findings suggest that mentions of health effects contributed the most in tweets with a positive hesitation score. Rage and discussions on misinformation became significantly higher (both *P*<.001) in the vaccination phase in India (Figure 3C), while an opposite trend was observed in the United States after approval on December 10, 2020 (Figure 3D) [37]. Similar analysis for the United Kingdom, Brazil, and Australia is shown in Figure S2 in Multimedia Appendix 2.

### Longitudinal Analysis Using an Alluvial Diagram

**Figure 4:**
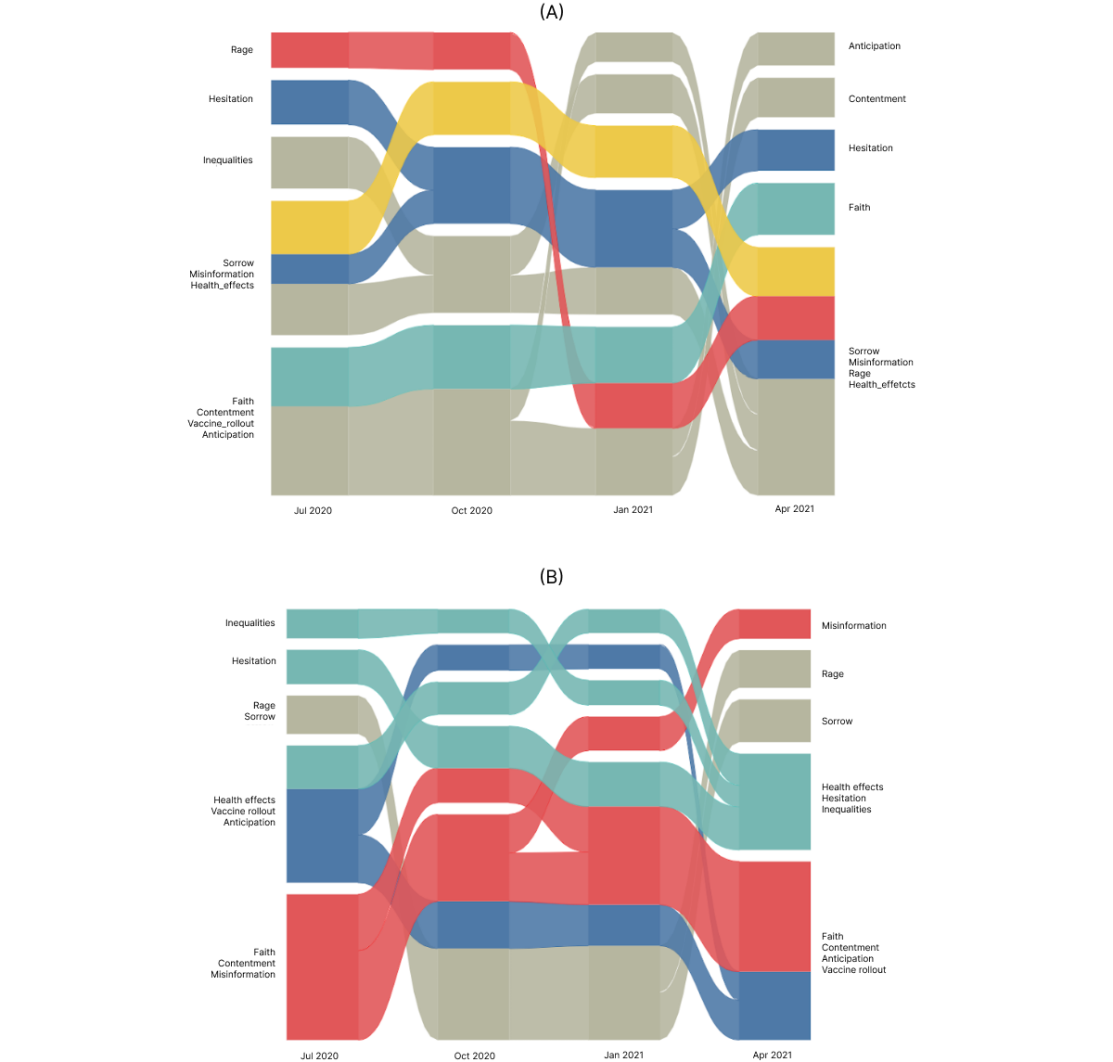
Alluvial diagram for correlation-based networks showing the evolution of categories from July 2020 to April 2021 at an interval of 3 months in (A) India and (B) the United States.

Inferences from the alluvial diagrams (Figure 4A) based on Infomap clustering on Pearson correlation networks demonstrated that all the influencing factors (ie, misinformation, health effects, inequities, and vaccine rollout) formed a primary module with emotions of sorrow and rage, which gained the highest PageRank in April 2021, the time when India saw the second wave of COVID-19 cases while the vaccine rollout continued. This articulates the stern sentiment of disappointment due to rising issues and the nonavailability of vaccines for people under the age of 45 years. It also had a high correlation with tweets mentioning the spread of misinformation. Faith, contentment, and anticipation, which were found to be highly associated in the early months of July 2020 and October 2020, were found to be relatively less important and unrelated in April 2021.

On the contrary, lexical categories representing positive sentiment in the United States evolved to a significant module. Faith, contentment, and anticipation toward the vaccine were found to have a positive correlation with each other (Figure 4B). Hesitation was the emotion influenced by mentions of health effects and inequities, whereas rage, sorrow, and misinformation were seen as less central factors in the United States.

**Figure 5:**
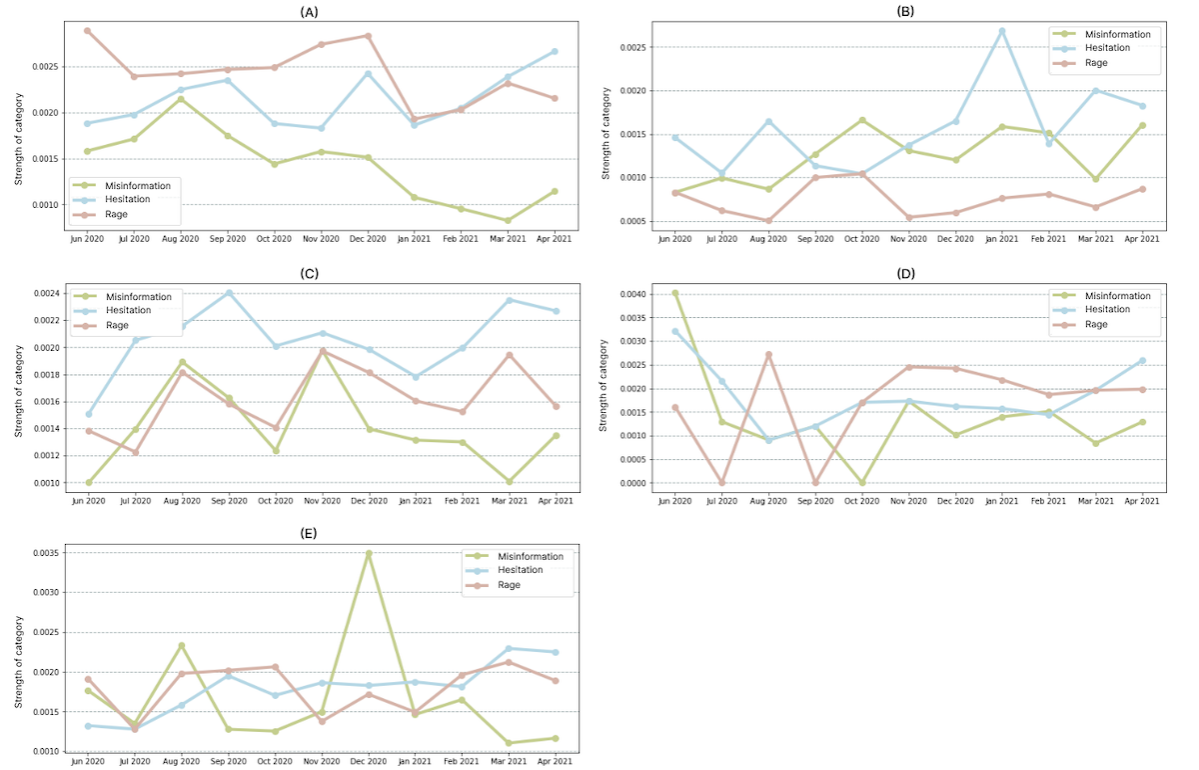
Comparing the temporal flow of strength of 3 categories (misinformation, hesitation, rage) for 5 countries: (A) United States, (B) India, (C) the United Kingdom, (D) Brazil, and (E) Australia.

Analysis of the temporal trend of misinformation, hesitation, and rage in the 5 countries is depicted in Figure 5. Updates regarding vaccinations started increasing near the end of 2020, which led to changing trends for hesitation expressed on Twitter. A notable inference from the line plots is that hesitation started rising from the beginning of 2021 when primary vaccination drives were initiated. In addition to this, rage is highly expressed in the tweets from the United States, while mentions of misinformation-related terms represented more significant proportions in India and the United Kingdom. Lexical categories of hesitation and rage were found to have similar trends, suggesting a tentative association between the 2 categories.

## Discussion

The rise in social media platforms, such as Twitter, has resulted in a valuable source to understand temporal variation in multiple affective and social categories. Influencing factors represented by word embedding–based lexical categories, namely misinformation, vaccine rollout, inequities, and health effects, significantly assisted in studying public perceptions toward emerging vaccine updates from initial approvals to rollout and administration.

### Principal Findings

Widespread misinformation being articulated through social media creates panic among users [38]. The misinformation category contains terms similar to “scam” and “conspiracy” from our data set that helped capture references of such words in the context of COVID-19 vaccines. High reporting of adverse effects and severe symptoms in rare cases leading to death [39] becomes a significant factor in increasing vaccination hesitation. The seed words given in the health effects category from the VAERS database led to the formation of its vocabulary containing “restless_sleep,” “skin_sensitivity,” “hot_flash,” “flulike_symptoms,” “complications,” and more. The semantic similarity-based approach allowed customization of categories according to our data set while ensuring the inclusion of rather noisy words like “feverish” and “achiness,” which cannot precisely be found in medical databases.

Inequalities based on socioeconomic status, religion, race, or demographics are standard in different countries, which can lead to inconsistencies while distributing vaccines. The inequities category encapsulated terms related to socioeconomic disparities and helped us identify the impact on other emotions. Based on inspection of our data set of tweets, we found words like “bigotry,” “underprivileged,” “financial_hardship,” and “institutional_racism” were occurring in a highly similar context toward vaccine distribution. Expression of inequities in April 2020 was found to be significantly anticorrelated with faith (*P*=.03) in India. Inaccessibility to vaccines in marginalized groups has led to lower gratification and higher anxiety among these groups [40].

We analyzed tweets from 5 countries belonging to different continents to get the generalized outlook toward vaccines and how they affect the global immunization process. Figure S3 in Multimedia Appendix 2 depicts sorrow, rage, and misinformation during April 2021 in the United Kingdom as the central module, with the highest PageRank. The Medicines and Healthcare products Regulatory Agency of the United Kingdom issued a new advisory during that period, concluding a possible link between AstraZeneca’s COVID-19 vaccine and extremely rare, unlikely occurrences of blood clots [41]. Upon a high-level investigation of the tweets from this period in the United Kingdom, we noticed that this press release had prompted multiple users to talk about blood clots due to the AstraZeneca vaccine. This could have been a potential contributing factor to the high strength of negative emotions expressed on social media platforms. Figure S4 in Multimedia Appendix 2 shows the alluvial diagram for Brazil. The category of rage, which was a relatively less important and independent module in the early months, had associations with sorrow and misinformation in April 2021 in Brazil. It aligned with a major peak in the numbers of cases and deaths during that period of the pandemic in Brazil [42]. In Figure S5 in Multimedia Appendix 2, we can see that faith, contentment, and vaccine rollout were relatively lower than other categories during July 2020, but later in April 2021, they formed a module with anticipation and gained the highest relative importance in the alluvial diagram. The announcement by the Australian government of securing an additional 20 million doses of the Pfizer-BioNTech COVID-19 vaccines overnight [43] happened in April 2021, and multiple tweets expressing optimism possibly contributed to the observed trend. Australia entered into 4 separate agreements with Pfizer, AstraZeneca, Novavax, and COVAX for the supply of COVID-19 vaccines, which resulted in a total number of approximately 170 million vaccine doses, as announced by the Prime Minister.

### Related Work

Existing literature on understanding vaccine hesitancy primarily focuses on defined questions from a part of the population belonging to a specific country [44-46]. Although such studies using surveys can help understand the explicit reasoning provided by the individuals, they still pose a limitation on inculcating the variation in outlooks of a larger population over a long period of time. We aimed to fill these gaps by studying important events, such as vaccine trials, highest reported deaths, or import and export of new vaccines, that fueled different populations’ emotions, as social media platforms are highly influential due to their comprehensive access and popularity. Our psychometric analysis considers important time stamps and a broader category of emotions to understand the before-after change and the factors with which they associate.

Identification of psychological processes that distinguish between vaccine-hesitant and receptive groups has been carried out in recent research [47]. This helps broadcast public health advisories on social media platforms by strategically taking into account the user's perspective. Effective public health interventions encouraging the uptake of COVID-19 vaccines have benefitted from psychologically oriented approaches [48,49].

Research around understanding the themes and general sentiments toward vaccination programs by analyzing social media posts has also been conducted [50,51]. Although their work provides an overview of positive, negative, or neutral sentiment around other important global developments affiliated with COVID-19 vaccine trials, our analysis provides intricate granularity in understanding the nature of emotions, temporal trends, and the influencing factors that have the highest correlations. Our pipeline effectively clusters the emotion categories and influencing factors around important time stamps based on vaccine approval with categories ranging from negative emotions like hesitation, rage, and sorrow to positive categories like contentment and faith. We further provide a framework to establish lexical categories for understanding the influencing factor correlation and its strength across crucial events. Identification of conspiracy theories related to COVID-19 vaccines has also been carried out [52], which can further be leveraged in addition to our work for improving the understanding of the underlying dynamics of social media posts and disrupting the spread of such content for improving vaccine uptake and tackling hesitancy.

### Limitations

Our study has some limitations. We extracted the tweets based on an empirical search of keywords and hashtags relevant to our study in “OR” combination with names of vaccines in the respective countries. Although this approach casts a wide net to retrieve tweets representing discourse around these vaccines, it does not guarantee that all posts were related to COVID-19 vaccine conversations specifically. The chosen keywords for the queries also might not include all relevant terms for capturing tweets specific to our objective. Our framework scores the emotions and influencing factors based on a normalized word count criteria and may miss nuanced language such as sarcasm. However, we interpreted our scores as the amount of discussion happening related to that category, such as hesitancy. Further, the selected categories for our framework are commonly identified emotions that indicate people’s perception toward vaccines. Our framework is designed to capture new categories and can be easily expanded and updated periodically to include relevant factors and emotion categories guided by contemporary patterns. Finally, a limitation of our study pertains to the representation bias inherent to social media–based analytics. However, considering that misinformation spreads the fastest through social media and we are considering trends, instead of absolute values, the results are expected to be fairly reliable. Future work may include segmentation of the trends by user demographics, and this information can help in developing tailored solutions for promoting inclusion of minority communities in campaigns. Vaccination drives and policies are targeted heavily toward older populations and minority groups that might not be an active part of such social media platforms. Therefore, for a better understanding of people’s opinions toward vaccines, further exploration via other mediums targeting various communities is essential.

### Conclusion

Our study provides research and practical implications for public policy making and research on vaccine hesitancy. Our findings offer insights into how the different stages of a pandemic and vaccination process influence emotions and crucial factors like misinformation, health discussions, and socioeconomic disparities on Twitter. This can help decision makers to navigate better solutions in future waves of COVID-19 or similar outbreaks and design appropriate interventions. Our approach can also be utilized to understand the general perception of people during such situations and what preventive measures should be implemented, taking the various influencing factors into account.

Future work can take the direction of local region-level analysis for a specific country to understand the granular emotions within different sections of people and the contributing factors behind them. Providing some weight to the number of reshares and likes the social media post gets can also play an essential role in including the influence the post had in calculating overall strength. Our approach has high adaptability and can be utilized for any online forum, news, or survey data to extract various insights. Designing categories and performing temporal analysis on social media data can also be used to identify multiple ongoing issues like the unavailability of medical resources like oxygen concentrators, intensive care unit beds, and drugs during the second wave of COVID-19. Such analysis can be taken into account while formulating quality allocation of scarce resources based on various factors and their strength. Better information extraction and understanding of such data can be facilitated through our work.

## Appendix

Multimedia Appendix 1 List of software and packages used for our study with their sources and identifiers for the reproducibility of this study.

Multimedia Appendix 2 Supplementary figures.

## Abbreviations

LIWC: Linguistic Inquiry and Word Count
NLTK: Natural Language Toolkit
VAERS: Vaccine Adverse Event Reporting System
