# Supplementary material for: Mining Trends of COVID-19 Vaccine Beliefs on Twitter With Lexical Embeddings: Longitudinal Observational Study
Source: JMIR Infodemiology. 2023 May 2;3:e34315. doi: 10.2196/34315 (PMC10165720; doi:10.2196/34315)
Supplement: Multimedia Appendix 1 [file infodemiology_v3i1e34315_app1.docx]

Supplementary Table 1. List of software and packages used for our study with their sources and identifiers for the reproducibility of this study.

| **Package/Library** | **Source** | **URL** |
| --- | --- | --- |
| Python (version 3.7.12) | python.org | <https://www.python.org/downloads/release/python-3712/> |
| nltk (version 3.2.5) | PyPI | <https://pypi.org/project/nltk/3.2.5/> |
| gensim (version 3.6.0) | PyPI | <https://pypi.org/project/gensim/3.6.0/> |
| networkx (version 2.5) | PyPI | <https://pypi.org/project/networkx/2.5/> |
| infomap (version 1.6.0) | PyPI | <https://pypi.org/project/infomap/1.6.0/> |
| pandas (version 1.1.5) | PyPI | <https://pypi.org/project/pandas/1.1.5/> |
| numpy (version 1.19.5) | PyPI | <https://pypi.org/project/numpy/1.19.5/> |
| scipy (version 1.4.1) | PyPI | <https://pypi.org/project/scipy/1.4.1/> |
| statsmodels (version 0.10.2) | PyPI | <https://pypi.org/project/statsmodels/0.10.2/> |
