# Supplementary material for: Mining Trends of COVID-19 Vaccine Beliefs on Twitter With Lexical Embeddings: Longitudinal Observational Study
Source: JMIR Infodemiology. 2023 May 2;3:e34315. doi: 10.2196/34315 (PMC10165720; doi:10.2196/34315)

Figure S1: Alluvial diagrams for Spearman correlation-based networks showing the evolution of categories from July 2020 to April 2021, on an interval of three months, in five countries, namely, (a) India, (b) USA, (c) UK, (d) Brazil, (e) Australia.

(a) India
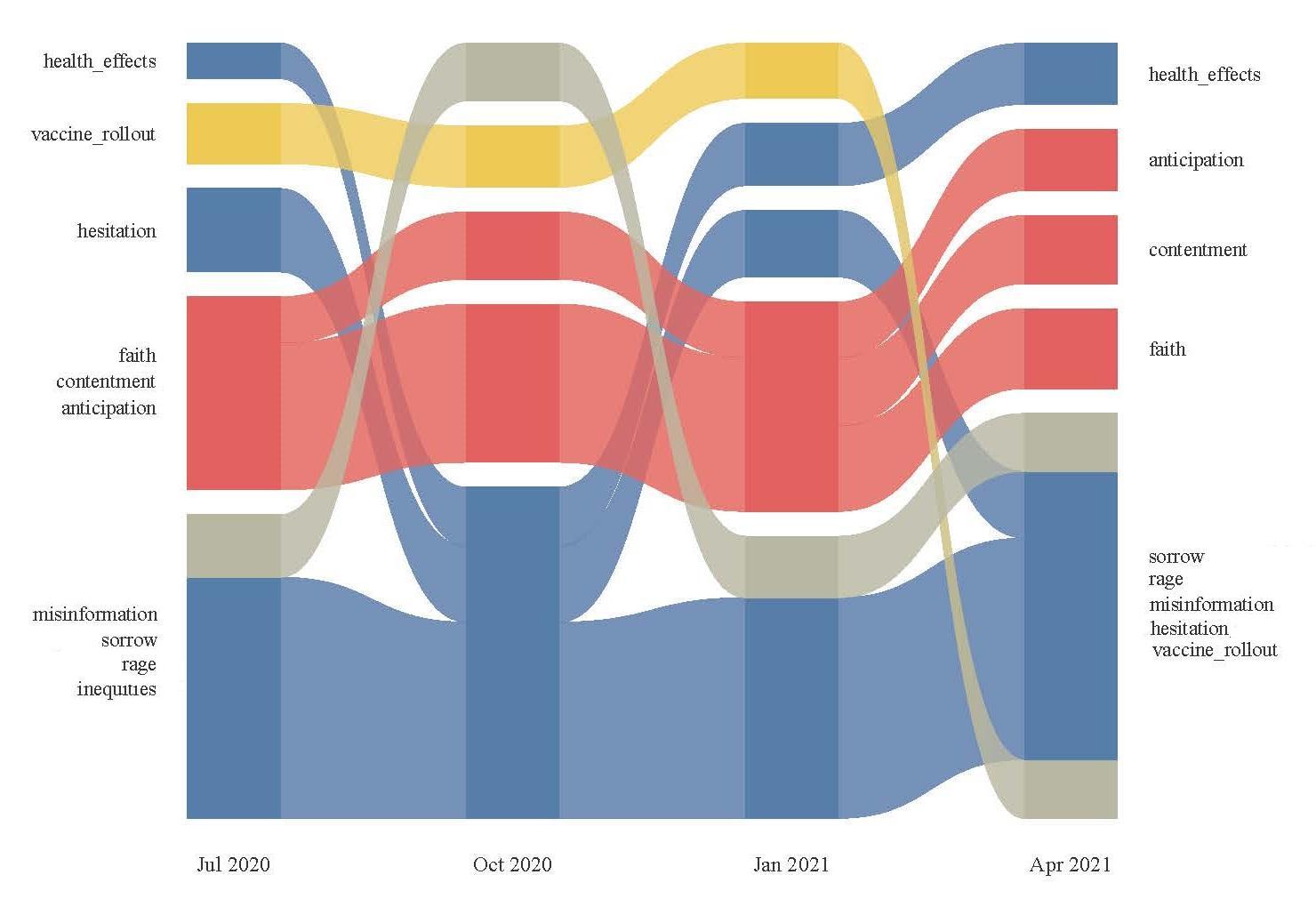


(b) USA


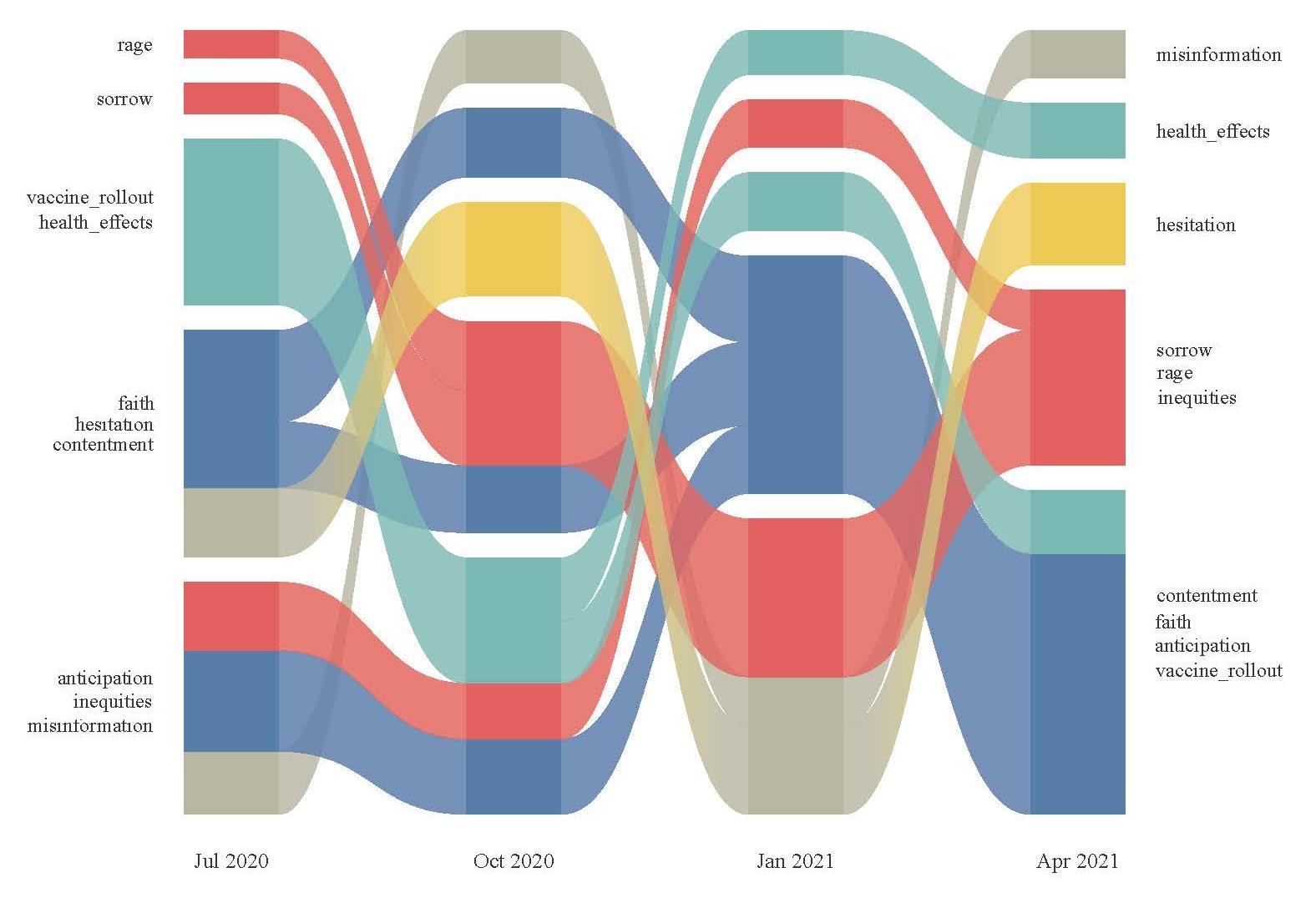


(c) UK


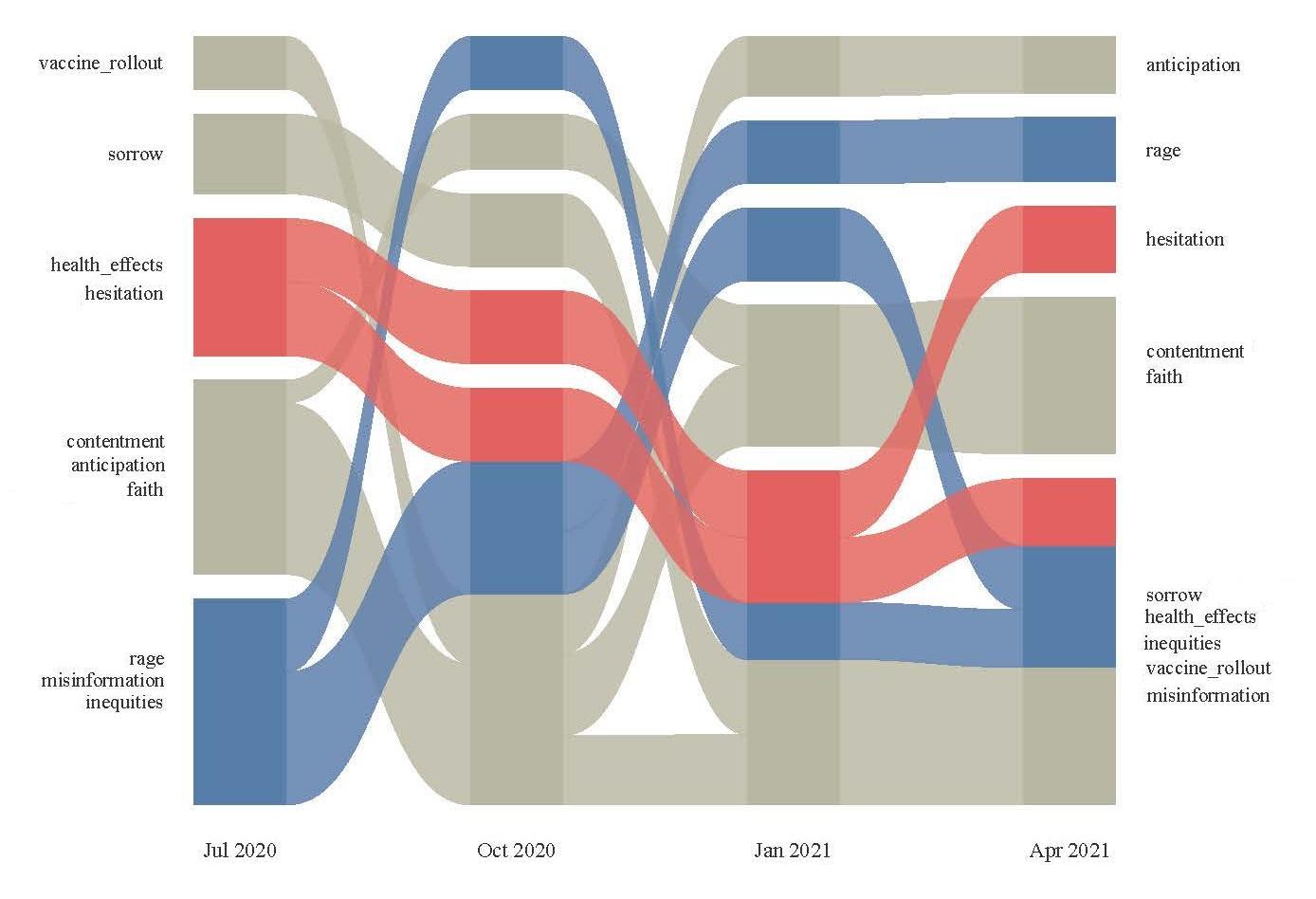


(d) Brazil


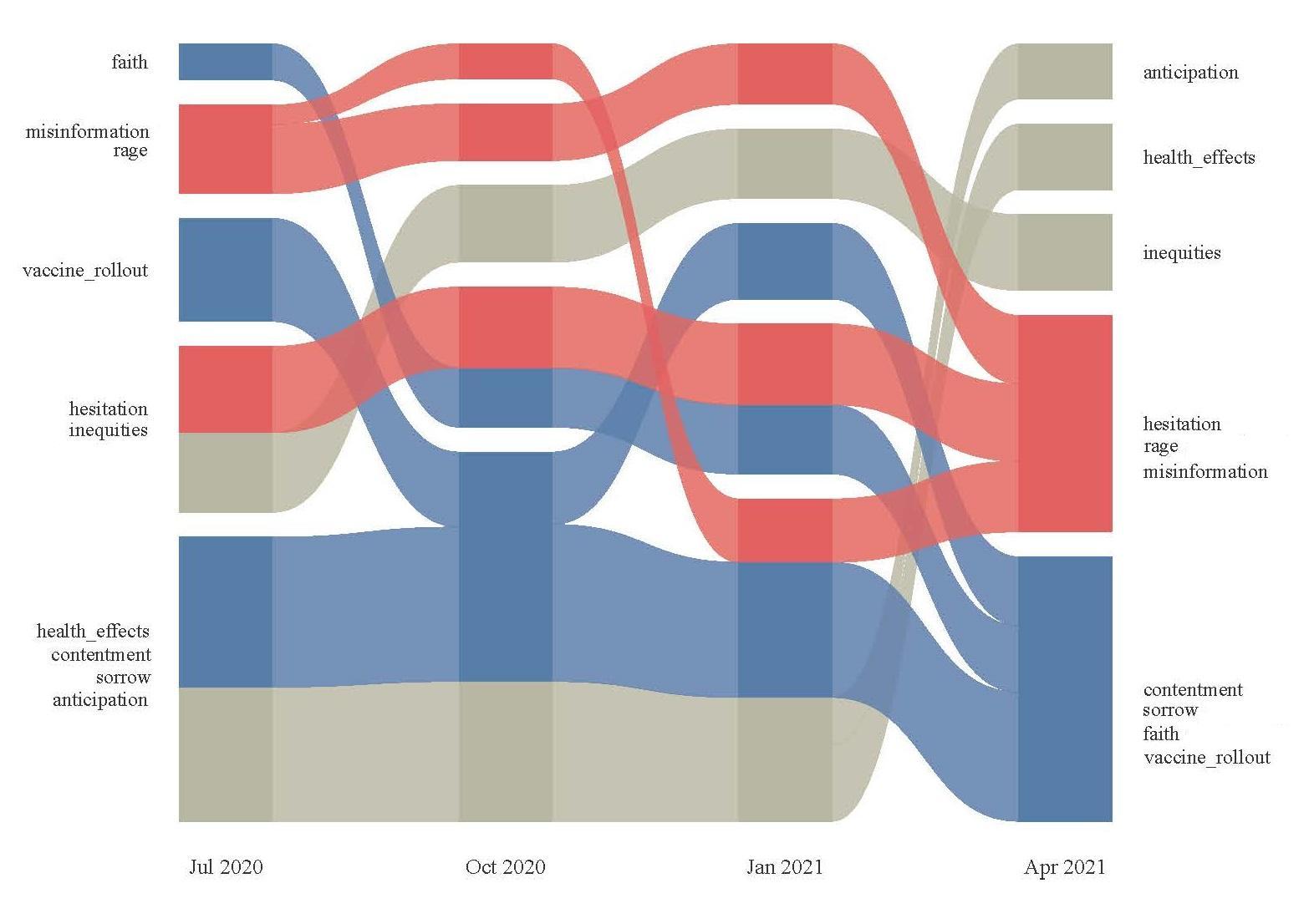


(e) Australia


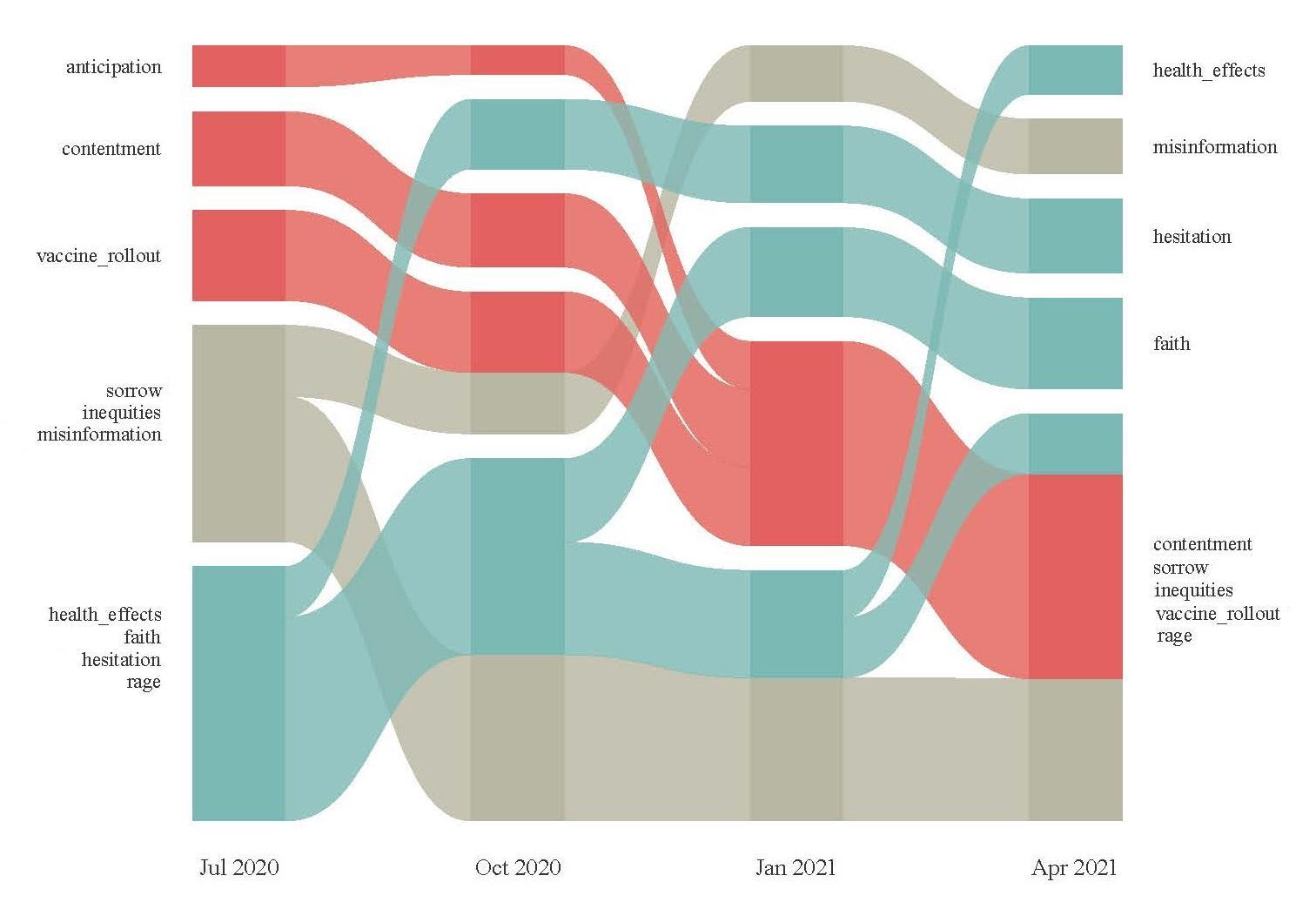


Figure S2: (Left): Percentage of tweets having a positive strength in each lexical category before and after approval of the COVID-19 vaccine in the United Kingdom, Brazil, and Australia. (Right): Percentage of Anticipation, Rage, Misinformation, Inequities, and Health Effects in positive ‘hesitancy’ tweets from UK, Brazil and Australia.


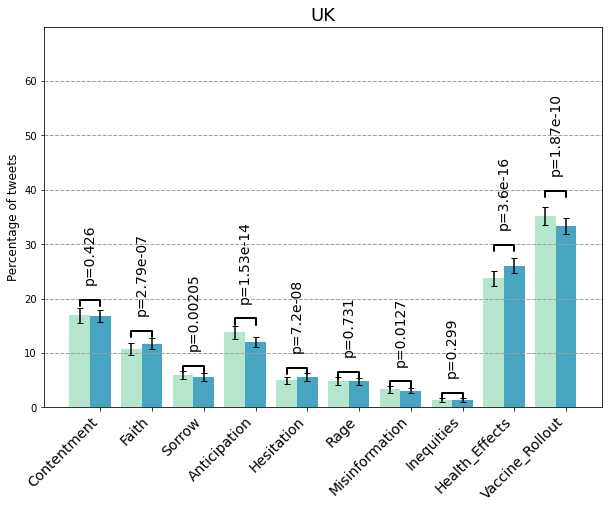

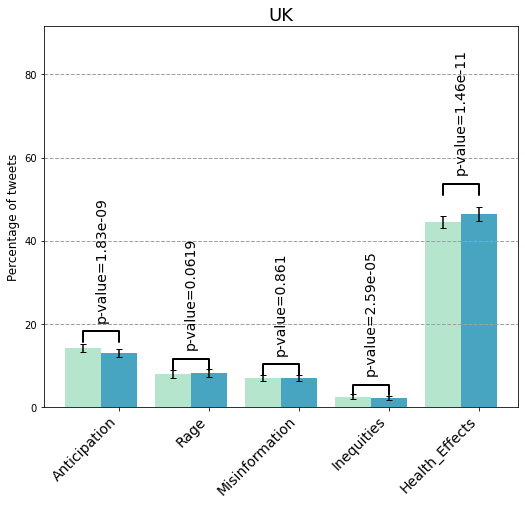


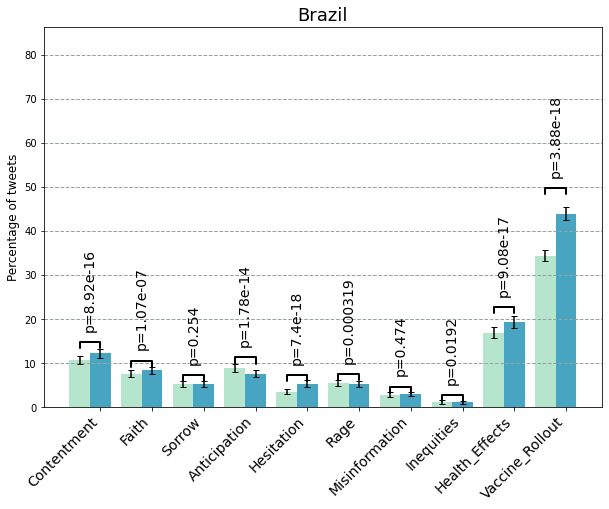

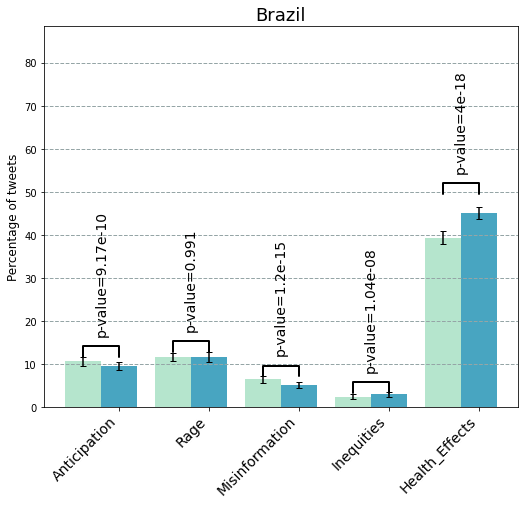


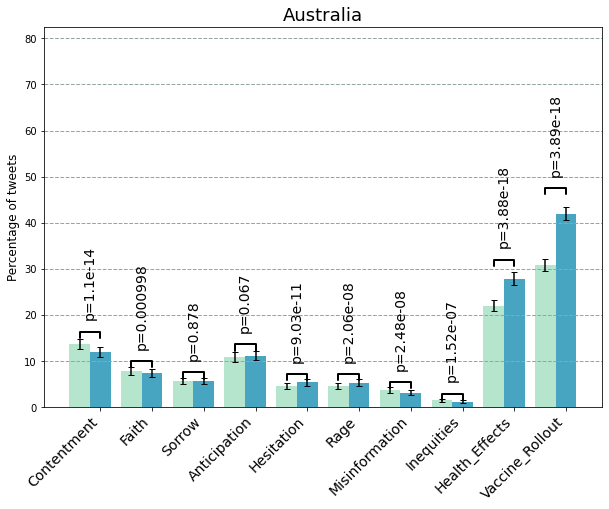

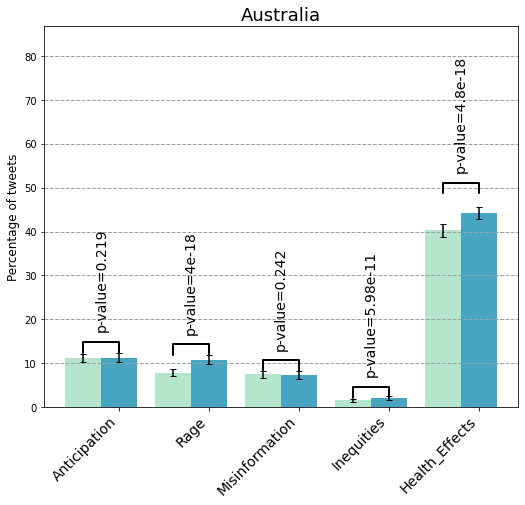


Figure S3: Alluvial diagram for correlation-based networks showing the evolution of categories from July 2020 to April 2021, on an interval of three months, in the UK.


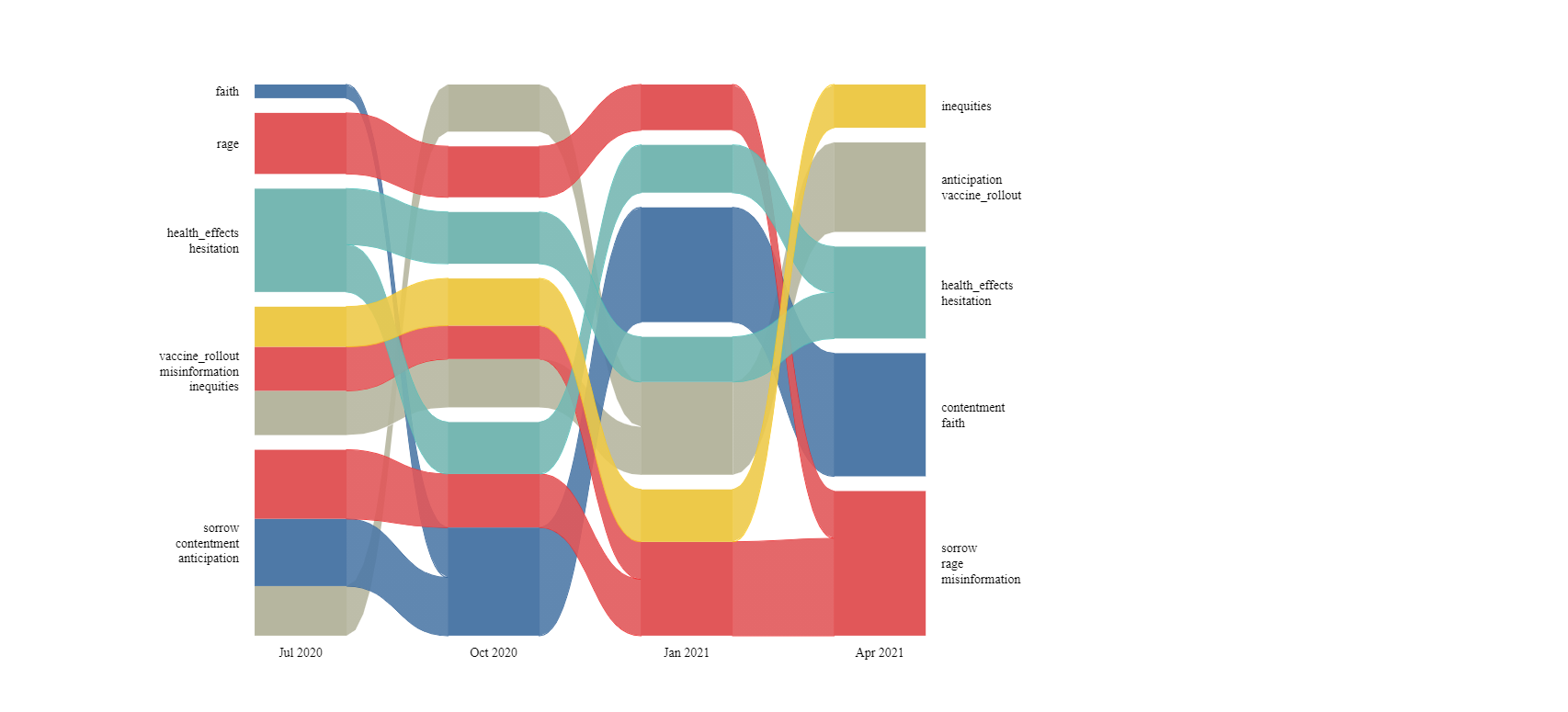


Figure S4: Alluvial diagram for correlation-based networks showing the evolution of categories from July 2020 to April 2021, on an interval of three months, in Brazil.


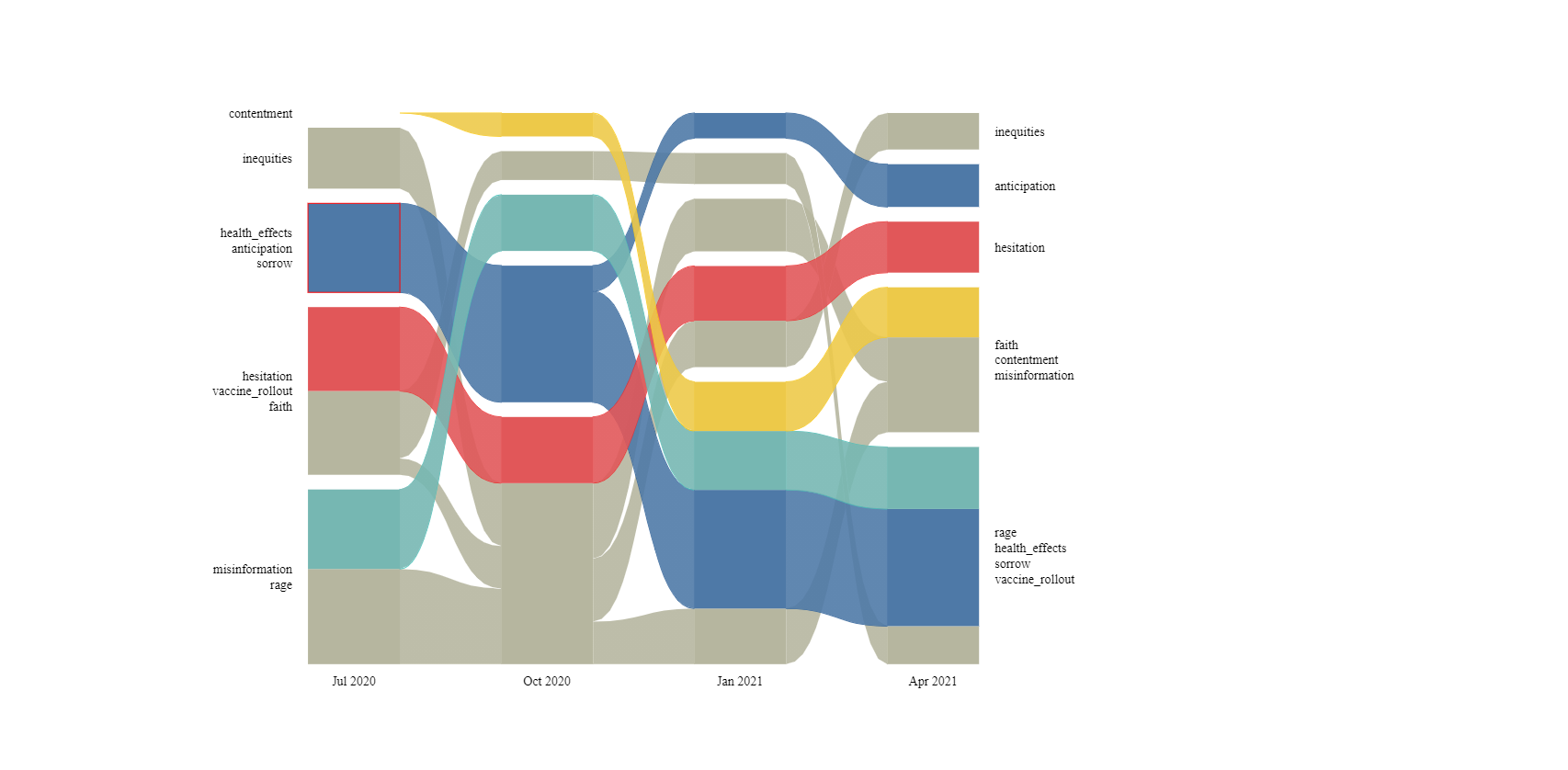


Figure S5: Alluvial diagram for correlation-based networks showing the evolution of categories from July 2020 to April 2021, on an interval of three months, in Australia.


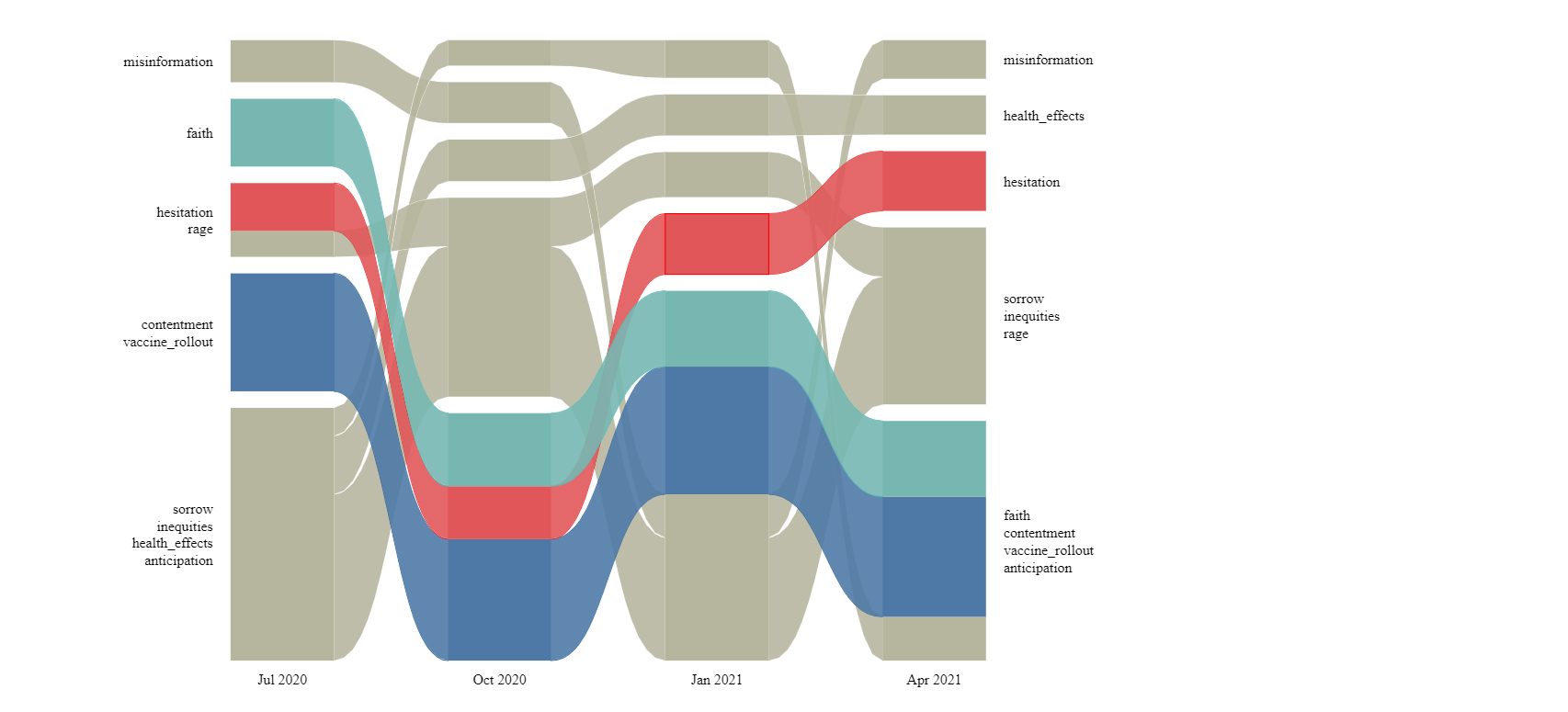

Supplement: Multimedia Appendix 2 [file infodemiology_v3i1e34315_app2.docx]
